# Persistent refractory hypercapnia following tracheostomy decannulation in a patient with brainstem hemorrhage: a case report

**DOI:** 10.3389/fresc.2026.1874164

**Published:** 2026-09-15

**Authors:** Yunhong Deng, Fuqiang Wang, Yaojiang Li, Dongzhen Xue, Jinfeng Liang, Hanbo Chen, Wenying Xie, Bizhou Fu, Man Hao

**Affiliations:** 1 Department of Rehabilitation Therapy, Guangdong Sanjiu Brain Hospital, Guangzhou, Guangdong, China; 2 Clinical College of Acupuncture and Rehabilitation, Guangzhou University of Chinese Medicine, Guangzhou, Guangdong, China; 3 Department of Rehabilitation Medicine, Guangdong Sanjiu Brain Hospital, Guangzhou, Guangdong, China; 4 Department of Radiology, Guangdong Sanjiu Brain Hospital, Guangzhou, Guangdong, China

**Keywords:** brainstem hemorrhage, case report, decannulation, hypercapnia, hypoventilation, tracheostomy

## Abstract

**Background:**

In tracheostomy decannulation decisions for patients with brainstem injury, clinicians typically focus on airway patency, swallowing, and overall respiratory status. However, some patients may remain at risk of chronic hypoventilation after decannulation because of impaired central ventilatory drive and other concomitant factors, even when anatomical airway patency has been restored.

**Case presentation:**

An elderly male with brainstem hemorrhage underwent decannulation after excision of granulation tissue in January 2024, with no immediate complications. During follow-up, he developed persistent carbon dioxide (CO₂) retention and was readmitted nine times for hypercapnic respiratory failure over the subsequent 19 months, with a peak arterial partial pressure of carbon dioxide of 172 mmHg. Despite repeated medical therapy and noninvasive ventilation, long-term ventilatory stability was not achieved, and a repeat tracheostomy was performed in August 2025.

**Conclusions:**

This case suggests that decannulation in brainstem-injury patients can be followed by a prolonged compensated phase—chronic compensated hypercapnia was clinically well tolerated for 19 months in this patient—before late, event-triggered decompensation. FEES performed during follow-up confirmed bilateral vocal cord paralysis with severe (penetration–aspiration scale level 7) aspiration, identifying a more direct mechanism for recurrent pneumonia and progressive ventilatory load than central drive impairment alone. The pre-decannulation PaCO₂ was normal, confirming that central ventilatory drive was sufficient at the time of decannulation; chronic compensated hypercapnia is not, in itself, an indication for recannulation. Longitudinal assessment of ventilatory stability is warranted, and the clinical course was likely multifactorial. Pre-decannulation FEES or flexible laryngoscopy should be considered in every brainstem-injury patient—not only those with documented dysphagia—and post-decannulation CO₂ monitoring should be targeted to selected high-risk patients in whom the results would alter management.

## 1 Introduction

Tracheostomy is essential for maintaining airway patency and providing ventilatory support in patients with severe brainstem hemorrhage. Decannulation decisions are based on airway patency, secretion management, cough effectiveness, swallowing and aspiration risk, and overall respiratory status. However, systematic reviews have shown that there are no universal standards or objective thresholds; in practice, decannulation often relies on expert opinion and clinical judgment, often emphasizing level of consciousness, swallowing function, and protective cough (1, 2). In neurocritical care and neurorehabilitation settings, decannulation is more complex, as neurogenic dysphagia, ineffective cough, excessive secretions, and altered airway anatomy and physiology may delay or complicate tracheostomy tube removal, underscoring the need for multidisciplinary, pathway-based management (3, 4). Weaning outcomes after acquired brain injury may vary according to lesion location, and infratentorial involvement has been associated with lower weaning success rates and greater long-term tracheostomy dependence (5).

The ventrolateral medulla and related brainstem networks generate respiratory rhythm and mediate chemoreception, thereby regulating ventilation in response to changes in arterial partial pressure of carbon dioxide (PaCO₂) and pH (6). Brainstem lesions, such as lateral medullary infarction, can cause acquired central hypoventilation, often manifesting as impaired spontaneous respiration during sleep, similar to ‘Ondine's curse,’ and accompanied by hypercapnia or recurrent respiratory failure (7–11). Damage to the medullary respiratory center or its associated networks can blunt or abolish the ventilatory response to elevated PaCO₂. Thus, even when the airway is anatomically patent, insufficient central ventilatory drive may impair carbon dioxide (CO₂) clearance and lead to persistent or recurrent hypercapnia (6, 9, 11). Brainstem lesions are also frequently accompanied by dysphagia and an increased risk of aspiration; subsequent aspiration pneumonia or respiratory infection can increase ventilatory demand and precipitate respiratory decompensation.

We report a case of brainstem hemorrhage in which the patient was initially stable after decannulation but subsequently developed persistent, refractory hypercapnia, experienced multiple episodes of severe clinical deterioration, and ultimately required repeat tracheostomy. This case highlights the clinical importance of prolonged CO₂ monitoring and optimized post-decannulation management in patients with brainstem injury.

## 2 Timeline

The clinical course is summarized below in chronological order; key arterial blood gas (ABG) values are provided in Table 1.

**Table 1:** Serial arterial blood gas measurements before and after tracheostomy decannulation.

Date	Clinical interpretation	pH	PaCO₂ (mmHg)	PaO₂ (mmHg)	HCO₃⁻ (mmol/L)	BE (mmol/L)
2024-01-03	Before decannulation	7.48	44.0	81	32.8	8.3
2024-07-01	Chronic compensated hypercapnia	7.41	65.0	71	42.2	14.9
2024-11-07	Chronic compensated hypercapnia	7.39	71.0	68	42.0	13.5
2024-11-12	Chronic compensated hypercapnia	7.39	61.0	82	37.5	10.8
2025-07-15	Chronic compensated hypercapnia	7.41	75.0	70	47.7	19.1
2025-07-24	Chronic compensated hypercapnia	7.42	63.0	68	41.5	14.8
2025-08-14	Worsening chronic hypercapnia	7.37	91.0	88	50.5	25.1
2025-08-18	Acute decompensation	7.32	131.9	97	66.1	40.1
2025-08-28	Severe acute decompensation before repeat tracheostomy	7.27	172.0	83	76.5	49.6
2025-08-29	Immediate post-intervention alkalemia	7.65	47.5	66	52.4	31.6
2025-09-11	Partial recompensation	7.47	48.5	53	34.9	11.2
2025-09-15	Partial recompensation	7.36	53.6	95	29.3	3.8
2025-10-31	Chronic compensated hypercapnia	7.38	71.0	68	42.0	13.5

ABG, arterial blood gas; PaCO₂, arterial partial pressure of carbon dioxide; PaO₂, arterial partial pressure of oxygen; HCO₃⁻, bicarbonate.

Acute phase (July 2023–January 2024). In July 2023, the patient—a previously independent elderly man—developed sudden impairment of consciousness and was diagnosed with brainstem hemorrhage. On 14 September 2023, he was transferred to our hospital while still comatose; tracheostomy and a nasogastric tube. Fiberoptic bronchoscopy on 27 December 2023 revealed proliferative granulation tissue above the tracheostomy tube, causing approximately 30% tracheal obstruction. A pre-decannulation ABG on 3 January 2024 showed PaCO₂ 44.0 mmHg without marked CO₂ retention, and the patient was judged ready for decannulation. On 8 January 2024, the granulation tissue was resected in the otolaryngology department and the tracheostomy tube was removed simultaneously. The decannulation was uneventful.

Subacute phase (February 2024–July 2025). The first readmission for ventilatory decompensation occurred in February 2024, at which point chronic compensated hypercapnia was identified. From February 2024 through August 2025, the patient was readmitted nine times for hypercapnic respiratory failure over a period of 19 months, with PaCO₂ chronically maintained in the 61.0–75.0 mmHg range. On 15 April 2024, follow-up neuroimaging identified a suspected hemorrhage in the splenium of the corpus callosum with intraventricular blood in the bilateral posterior horns of the lateral ventricles; serial imaging throughout the subsequent 17 months suggested mild progressive enlargement of the lateral ventricles and the fourth ventricle compared with the pre-event baseline (July 2023), findings compatible with—but not diagnostic of—secondary communicating hydrocephalus.

Acute-on-chronic decompensation (August 2025). On 14 August 2025, PaCO₂ increased to 91.0 mmHg. The patient then experienced a first acute decompensation on 18 August (PaCO₂ 131.9 mmHg with acidemia), followed by a second, more severe decompensation on 28 August (PaCO₂ 172.0 mmHg), prompting repeat tracheostomy on the same day. At the final follow-up in October 2025, respiratory status had improved, with PaCO₂ returning to the chronic compensated range; however, no neurological improvement was observed.

## 3 Patient information

### 3.1 Patient demographics and baseline characteristics

An elderly man presented with sudden impairment of consciousness in July 2023 and was diagnosed with brainstem hemorrhage. Baseline risk profile included grade 3 hypertension (very high risk category), body mass index of 20.5 kg/m^2^, no smoking history, no known chronic lung disease, and no sedative or opioid exposure.

### 3.2 Clinical context at transfer

Upon transfer to our hospital on 14 September 2023, the patient remained comatose. Consciousness was assessed using the Glasgow Coma Scale (GCS), with a score of 7 (E4VTM3). He required tracheostomy and insertion of a nasogastric tube.

### 3.3 Clinical findings neurological assessment

The patient developed prolonged disorder of consciousness following intracerebral hemorrhage, characterized by unresponsiveness, occasional automatic eye opening, and poor response to painful stimuli. Residual neurological deficits included chronic respiratory failure due to compromised central respiratory drive and weak cough reflex, and generalized limb muscle atrophy from prolonged immobilization and motor dysfunction.

### 3.4 Swallowing and airway protection

Swallowing function was assessed using the Standardized Swallowing Assessment (SSA), with a score of 45, indicating severe dysphagia. The absence of an effective spontaneous cough indicated markedly impaired airway protection.

### 3.5 Pulmonary status at follow-up

At the final follow-up in October 2025, chest CT showed atelectasis with superimposed infection and a small bilateral pleural effusion.

### 3.6 Diagnostic assessment brain imaging

Brain computed tomography (CT) performed at initial onset in July 2023 confirmed hemorrhage confined to the midbrain and pons, with no involvement of the upper medulla (Figure 1a). The original CT obtained at the referring hospital on 11 July 2023 showed well-defined hypodense areas in the pons and midbrain (Figure 1b). Neuroimaging on 15 April 2024 revealed a suspected hemorrhage in the splenium of the corpus callosum with intraventricular blood in the bilateral posterior horns of the lateral ventricles. Compared with the pre-event baseline (July 2023), serial CT scans obtained over the subsequent 17 months suggested mild enlargement of the lateral ventricles and the fourth ventricle (Figure 2). These findings are compatible with secondary communicating hydrocephalus following IVH, but the diagnosis is descriptive rather than quantitative: formal metrics (Evan's index, callosal angle) and CSF opening pressure were not consistently documented, and no transependymal CSF migration or radiological signs of acute obstructive hydrocephalus were observed.

**Figure 1:**
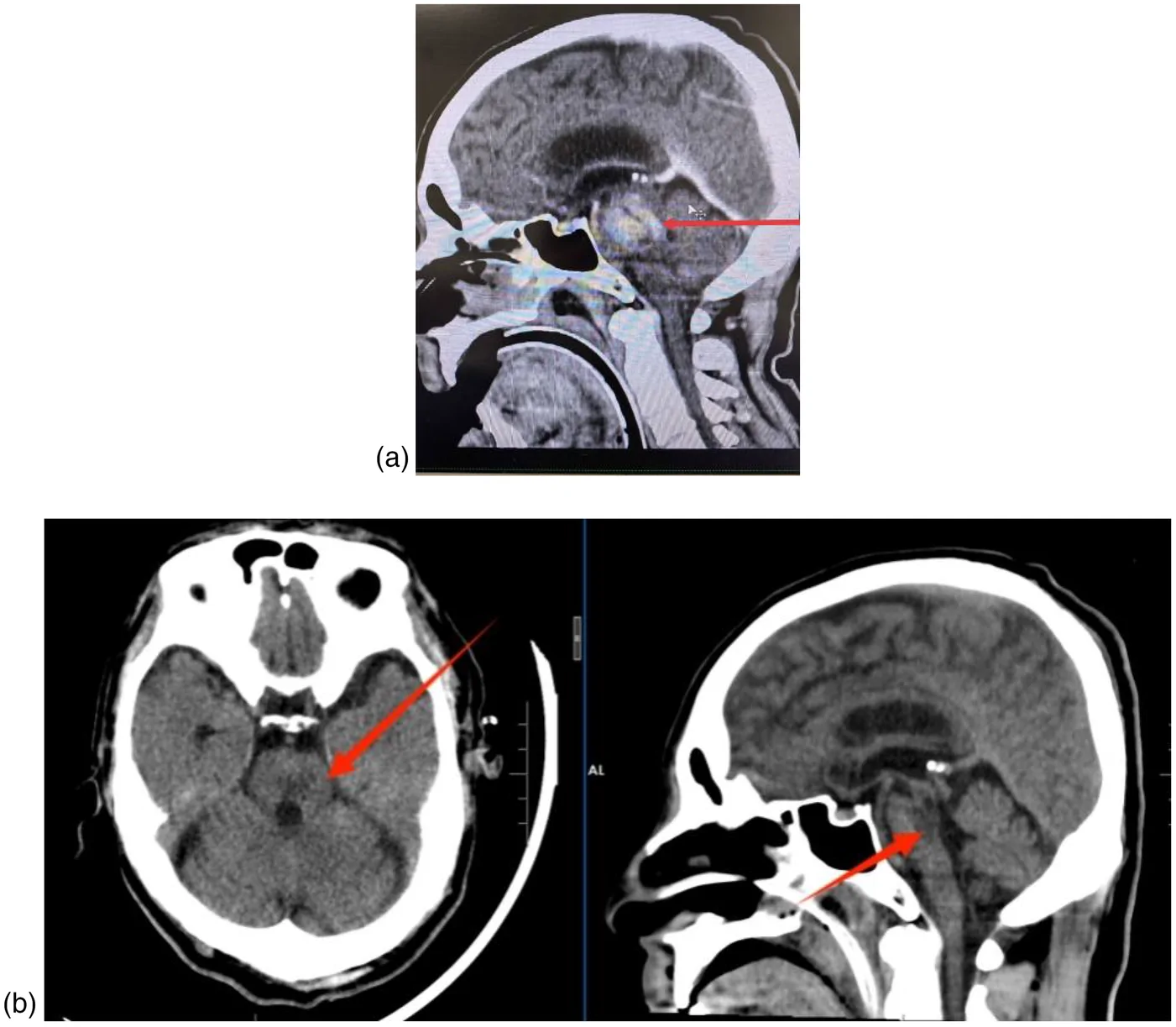
**(a)** CT performed at initial onset in July 2023 confirmed hemorrhage confined to the midbrain and pons. **(b)** CT performed on 11 July 2023, showed well-defined hypodense areas in the pons and midbrain.

**Figure 2:**
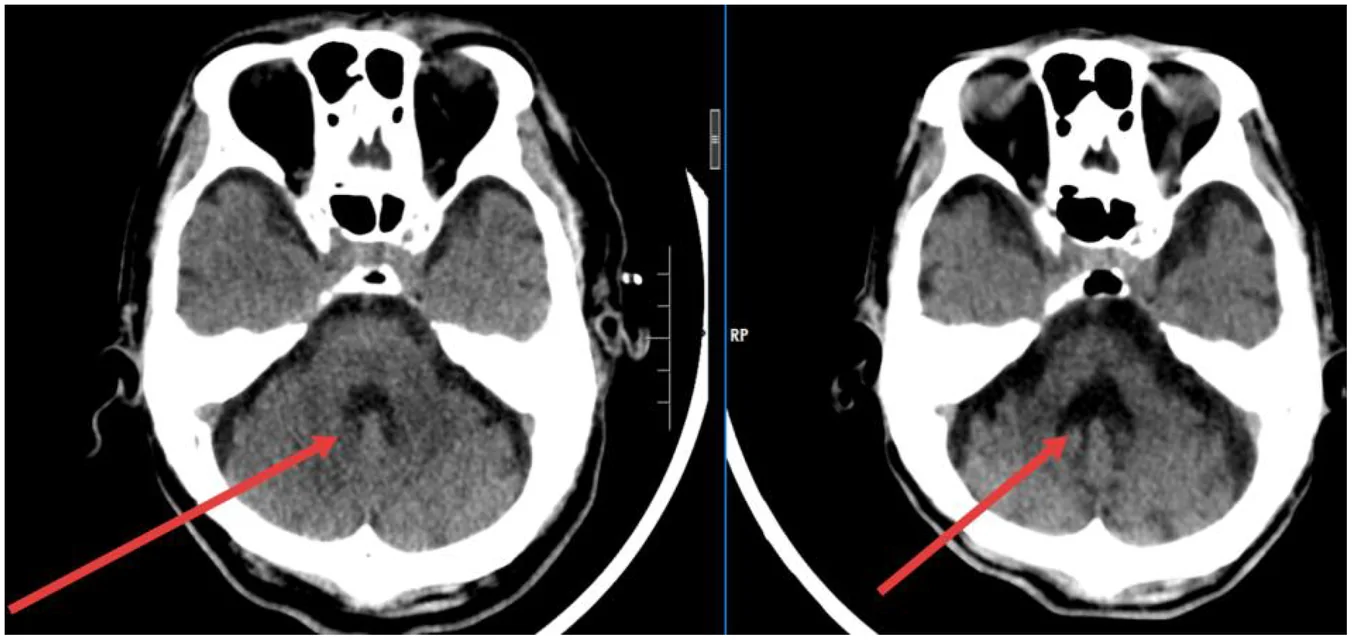
Serial cranial imaging comparison. Left panel: imaging acquired on 13 September 2023; right panel: imaging acquired on 23 April 2026. Mild interval enlargement of bilateral lateral ventricles and the fourth ventricle is noted, radiographically suggestive of hydrocephalus.

### 3.7 Bronchoscopy

To evaluate the feasibility of decannulation, fiberoptic bronchoscopy was performed on 27 December 2023, revealing proliferative granulation tissue above the tracheostomy tube causing approximately 30% obstruction of the tracheal lumen (Figure 3). Airway secretions were considered manageable, with suctioning required approximately twice daily.

**Figure 3:**
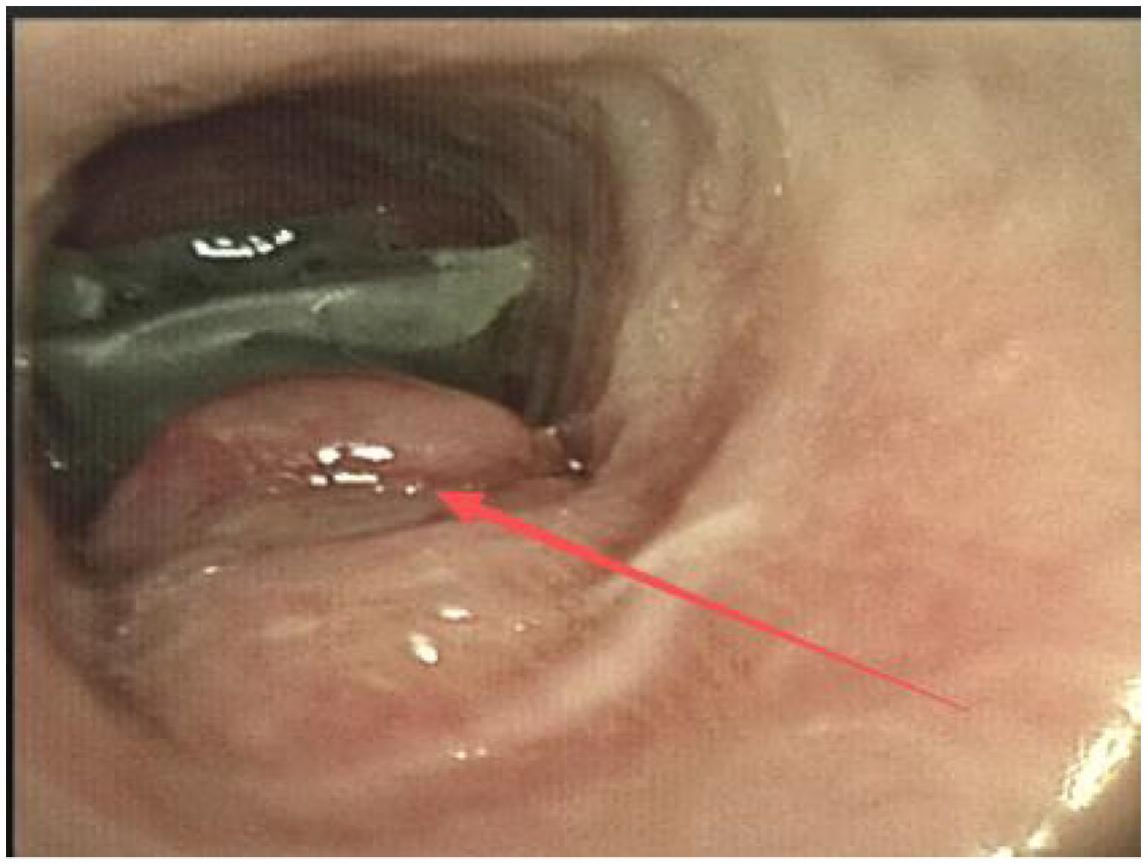
Fibreoptic bronchoscopy (27 December 2023) showed proliferative granulation tissue above the tracheal tube, obstructing approximately 30% of the tracheal lumen.

Flexible laryngoscopy and fiberoptic endoscopic evaluation of swallowing (FEES). Flexible laryngoscopy combined with FEES was performed on 28 February 2025 (approximately 13.5 months after decannulation). Laryngoscopic examination of the laryngeal vestibule showed poor visualization of the glottic inlet, with the bilateral arytenoid cartilages being normal in appearance without swelling (Figure 4A). Pharyngeal motor activity was markedly abnormal, with involuntary palatopharyngeal tremor and circumferential contraction of the pharyngeal muscles during inspiration, with bilateral adduction toward the tongue base (Figure 4B). Pooled secretions were observed within the laryngeal vestibule, consistent with Murray secretion scale grade 2 (Figure 4C). Both vocal cords were fixed in a semi-open resting position, with persistent glottic closure insufficiency (Figure 4D). The pharyngeal reflex was blunted, the cough reflex was delayed (with only partially effective cough clearance), and compensatory effortful swallowing was ineffective. Both vallecular and pyriform sinus residues were graded as severe, and the Penetration–Aspiration Scale (PAS) level was 7 (food entered the airway below the level of the vocal folds and could not be cleared even with effort). The overall FEES impression was oropharyngeal dysphagia. Collectively, these findings are consistent with a central (neurogenic) motor disorder involving brainstem sensorimotor control of swallowing and airway protection, supporting a diagnosis of acquired vagal neuropathy secondary to brainstem injury, without evidence of structural upper airway malformation (Figure 4).

**Figure 4:**
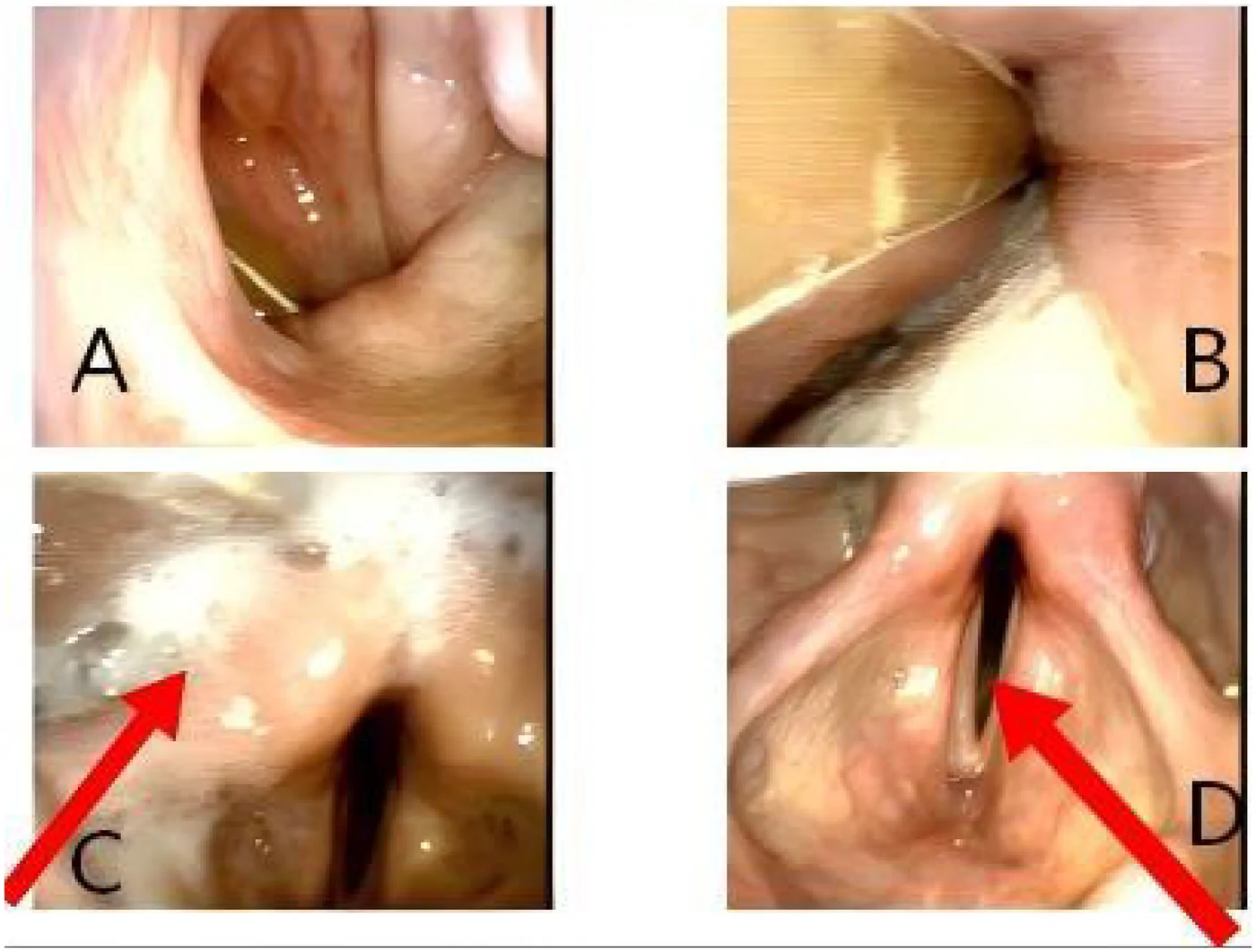
**(A)** flexible laryngoscopy combined with FEES was performed on 28 February 2025 (approximately 13.5 months after decannulation). Laryngoscopic examination of the laryngeal vestibule showed poor visualization of the glottic inlet, with the bilateral arytenoid cartilages being normal in appearance without swelling. **(B)** Pharyngeal motor activity was markedly abnormal, with involuntary palatopharyngeal tremor and circumferential contraction of the pharyngeal muscles during inspiration, with bilateral adduction toward the tongue base. **(C)** Pooled secretions were observed within the laryngeal vestibule, consistent with Murray secretion scale grade 2. **(D)** Both vocal cords were fixed in a semi-open resting position, with persistent glottic closure insufficiency. The pharyngeal reflex was blunted, the cough reflex was delayed (with only partially effective cough clearance), and compensatory effortful swallowing was ineffective. Both vallecular and pyriform sinus residues were graded as severe, and the Penetration–Aspiration Scale (PAS) level was 7 (food entered the airway below the level of the vocal folds and could not be cleared even with effort).

### 3.8 Bedside assessment of upper-airway patency on 28 February 2025

On 28 February 2025, during the same session in which FEES was performed, bedside clinical assessment captured the following cardinal signs of upper-airway obstruction (Supplementary Video S1): inspiratory dyspnea with three-concavity sign (suprasternal, supraclavicular, and intercostal retractions); accessory breathing muscle recruitment; stridor; tachypnea with open-mouth breathing; and nasal cannula oxy gen supplementation. In the diagnostic framework previously proposed (1), the combination of stridor, accessory muscle use, and dyspnea identifies bilateral vocal cord paralysis, aspiration, and weak cough as the leading cause of respiratory pump failure—a pattern that is fully consistent with the FEES-documented bilateral vocal cord immobility and PAS level 7 in this patient.

### 3.9 Arterial blood gas monitoring

Serial arterial blood gas analyses demonstrated chronic compensated hypercapnia after decannulation, with PaCO₂ most commonly ranging from 61.0 to 75.0 mmHg, accompanied by near-normal pH and elevated bicarbonate levels. Detailed results are summarized in Table 1. The overall longitudinal clinical course is shown in Figure 5. In August 2025, PaCO₂ rose to 91.0 mmHg (14 August), then reached 131.9 mmHg (18 August) and 172.0 mmHg (28 August), both with acidemia.

**Figure 5:**
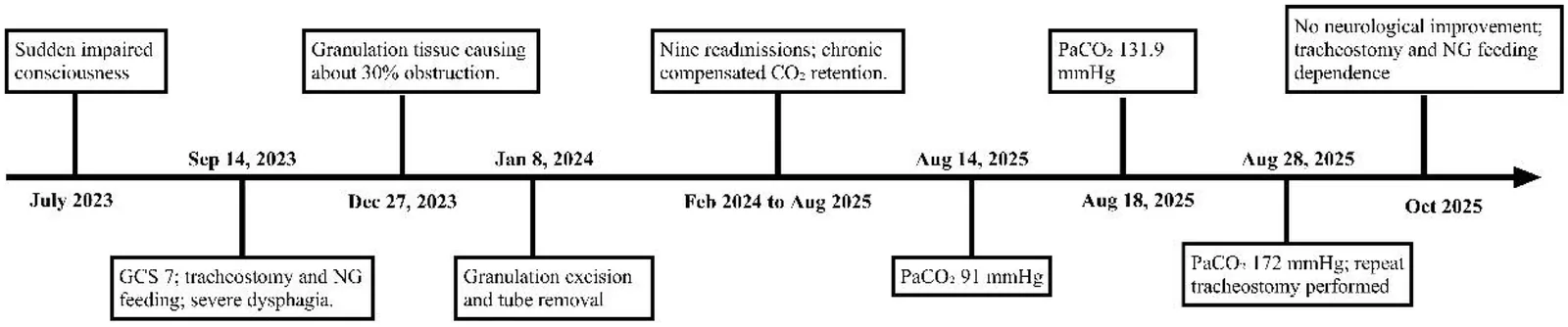
Timeline of the patient's clinical course.

### 3.10 Ventilation assessment

During multiple hospitalizations, the patient received standard medical management, including antibiotics, airway care, and non-invasive bilevel positive airway pressure (BiPAP) ventilation. Specifically, a ResMed Stellar 100 ventilator was utilized in ST mode with the following settings: IPAP 17.0 cmH₂O, EPAP 5.0 cmH₂O, and backup respiratory rate 20 breaths per minute; real-time monitoring showed a respiratory rate of 20 breaths per minute, tidal volume of 710 mL, and minute ventilation of 14.2 L/min. A Mindray SV650 ventilator in pressure-controlled synchronized intermittent mandatory ventilation (P-SIMV) mode was also employed. Despite progressive optimization of ventilation parameters, CO₂ retention persisted, with PaCO₂ rising progressively to 172 mmHg. Given that continuous nocturnal oxygen saturation or carbon dioxide monitoring, polysomnography, and systematic assessment of diaphragmatic or neuromuscular function were not consistently available, isolated central hypoventilation could not be confirmed definitively.

### 3.11 Therapeutic interventions management of airway obstruction

After multidisciplinary evaluation, the team considered that the mechanical airway obstruction should be removed before decannulation. On 8 January 2024, the granulation tissue was resected in the otolaryngology department, and the tracheostomy tube was removed concurrently with no acute complications. A pre-decannulation arterial blood gas analysis performed on 3 January 2024 showed no marked carbon dioxide retention, with a PaCO₂ of 44.0 mmHg.

### 3.12 Management of post-decannulation hypercapnia

Although conservative measures—including BiPAP ventilation, airway care, and antibiotics—achieved temporary clinical improvement, long-term ventilatory stability could not be sustained. Given recurrent life-threatening hypercapnia, a repeat tracheostomy was performed on 28 August 2025.

### 3.13 Follow-up and outcomes

During the 19 months following decannulation (February 2024–August 2025), the patient was readmitted nine times for ventilatory decompensation. Repeated evaluation suggested that although the mechanical airway problem had been corrected, sustained ventilatory stability had not been achieved. Given the pontine-midbrain lesion, impairment of central ventilatory drive was strongly suspected. Nevertheless, the overall clinical course was likely multifactorial (Table 2). Severe neurological impairment, prolonged immobility, persistent dysphagia, ineffective airway protection, and recurrent ventilatory burden related to aspiration or infection may also have contributed to chronic hypoventilation.

**Table 2:** Graded multifactorial analysis of contributing factors in this case.

Contributing factor	Evidence in this case
Bilateral central vocal cord paralysis (acquired vagal neuropathy secondary to brainstem injury)	Confirmed via FEES performed on 28 February 2025, showing bilateral vocal cord immobility at a semi-open resting position
Severe oropharyngeal dysphagia graded as Penetration–Aspiration Scale level 7	Confirmed on FEES conducted on 28 February 2025
Recurrent aspiration pneumonia	Clinically confirmed, with infectious episodes documented during multiple hospital readmissions
Prolonged immobility and respiratory muscle weakness	Robust clinical evidence: 19 months of bed confinement accompanied by generalized limb muscular atrophy
New-onset intracerebral hemorrhage with intraventricular extension (April 2024, splenium of the corpus callosum)	Radiographically documented on cranial neuroimaging
Imaging findings suggestive of secondary communicating hydrocephalus	Neuroimaging features compatible with hydrocephalus, though insufficient for definitive diagnosis
Intercurrent infection precipitating late clinical decompensation	Probable etiology for clinical deterioration observed in August 2025
Impaired central ventilatory drive secondary to midbrain–pontine lesions	Biologically plausible yet not quantitatively assessed; pre-decannulation PaCO₂ remained within the normal range
Grade 3 chronic hypertension	Well-documented medical history; autonomic nervous system involvement was not formally evaluated
Obesity and BMI-related risk factors	Excluded (BMI = 20.5 kg/m^2^, within normal range)
Pre-existing chronic pulmonary disease	Excluded (no relevant medical history)
Sedative or opioid medication exposure	Excluded (no documented administration history)
GLP-1 receptor agonist medication use	Excluded (no relevant medication record)
Congenital upper airway malformations	Excluded via FEES; vocal cord dysfunction was confirmed as acquired central neuropathy

FEES; fiberoptic endoscopic evaluation of swallowing; PaCO₂, arterial partial pressure of carbon dioxide; BMI, body mass index.

### 3.14 Post-tracheostomy outcome

Following the repeat tracheostomy on 28 August 2025, respiratory status improved, and arterial blood gas parameters normalized to levels consistent with chronic compensated hypercapnia.

### 3.15 Final follow-up (October 2025)

At the final follow-up in October 2025, no neurological improvement was observed. The patient remained comatose with a GCS score of 7 (E4VTM3), continued to have severe dysphagia with an SSA score of 45, and remained dependent on tracheostomy and a nasogastric tube. Chest CT showed atelectasis with superimposed infection and a small pleural effusion.

## 4 Discussion and conclusions

This case highlights that anatomical readiness for decannulation does not necessarily indicate sustained ventilatory readiness in patients with brainstem injury. After granulation tissue removal and apparently successful decannulation following airway reassessment and a 24-hour tracheostomy capping trial, this patient developed persistent compensated chronic hypercapnia and was readmitted nine times for acute-on-chronic hypercapnic respiratory failure over the subsequent 19 months, ultimately requiring repeat tracheostomy. The clinical course suggests that post-decannulation success in such patients should be judged not only by airway patency, secretion burden, and swallowing status, but also by long-term ventilatory stability. Notably, the 17-month chronic compensated phase demonstrates that decannulation can be sustained for an extended period when compensatory mechanisms are maintained, and that the late decompensation was event-triggered rather than a manifestation of progressive central drive failure. The pre-decannulation PaCO₂ was normal (44.0 mmHg), confirming that central ventilatory drive was sufficient at the time of decannulation.

Impaired central ventilatory drive was strongly suspected in this patient during follow-up, although the pre-decannulation PaCO₂ was normal. The pontine-midbrain lesion may have blunted carbon dioxide chemosensitivity and ventilatory responses, thereby predisposing the patient to chronic carbon dioxide retention during the subsequent post-decannulation period and subsequent respiratory decompensation during sleep or intercurrent illness (6). However, the clinical trajectory was likely multifactorial rather than driven by a single mechanism. Importantly, the bedside clinical signs documented on 28 February 2025, during the same session as FEES—specifically, inspiratory dyspnea with three-concavity sign (suprasternal, supraclavicular, and intercostal retractions), accessory breathing muscle recruitment, stridor, and tachypnea—indicate upper-airway obstruction rather than central hypoventilation in isolation. the combination of stridor, accessory muscle use, and dyspnea identifies bilateral vocal cord paralysis, aspiration, and weak cough as the leading cause of respiratory pump failure, which is fully consistent with the FEES-documented bilateral vocal cord immobility in a semi-open resting position and penetration–aspiration scale level 7 in this patient. Computed tomography at the initial onset confirmed hemorrhage confined to the midbrain and pons, with no involvement of the upper medulla (Figure 1a); therefore, an undiagnosed medullary lesion is not a likely explanation. The 13.5-month interval between decannulation and FEES implies that severe aspiration persisted throughout the post-decannulation phase, exerting cumulative effects that culminated in late respiratory decompensation. This observation highlights the value of integrating both bedside assessment of upper-airway patency (inspection for inspiratory stridor, accessory muscle recruitment) and FEES into routine pre-decannulation evaluations for patients with brainstem injury, rather than relying on either modality alone.

This case also underscores a practical gap in post-decannulation follow-up. In this patient, neither end-tidal carbon dioxide nor transcutaneous carbon dioxide monitoring was used after decannulation, which may have delayed the recognition of occult or sleep-related hypoventilation. Previous studies have shown that transcutaneous carbon dioxide correlates reasonably well with arterial carbon dioxide in chronic hypoventilation and in patients receiving non-invasive ventilation, and may be more informative than end-tidal carbon dioxide in some non-intubated settings (12, 13). Although formal monitoring protocols cannot be established based on a single case, this clinical experience suggests that scheduled carbon dioxide monitoring may benefit selected high-risk patients following decannulation — particularly those with brainstem injury, persistent severe neurological impairment, FEES-documented severe aspiration, recurrent aspiration risk, or unexplained hospital readmissions. However, the present case does not support routine universal carbon dioxide monitoring after decannulation; surveillance should be targeted exclusively to high-risk individuals and undertaken only when results are expected to guide changes in clinical management. Chronic compensated hypercapnia with preserved pH, in the absence of clinical deterioration, does not constitute an independent indication for ongoing monitoring or re-tracheostomy.

Pre- vs. post-decannulation clinical and physiological comparisons provide additional insight into the candidate mechanisms in this patient. Pre-decannulation, the patient had a normal PaCO₂ (44.0 mmHg) on 3 January 2024 and showed no clinical signs of central apnoea, hypothalamic–pituitary dysfunction, or classical Weber's syndrome, arguing against a clinically manifest central ventilatory, autonomic, or brainstem somatic motor syndrome at the time of decannulation. Post-decannulation, three distinct phases were observed: (a) a 17-month chronic compensated phase with stable PaCO₂ of 61.0–75.0 mmHg and preserved pH, indicating that compensatory ventilatory mechanisms (renal bicarbonate retention, sustained wakeful drive) were sufficient to maintain acid–base homeostasis; (b) an acute decompensation phase in August 2025, when PaCO₂ escalated from 91.0 to 172.0 mmHg, coinciding with the FEES-documented bilateral central vocal cord paralysis, severe (penetration–aspiration scale level 7) aspiration, and recurrent pneumonia, identifying these as the principal drivers of late decompensation; and (c) a post-re-tracheostomy phase with partial recompensation. This pre/post-treatment comparison supports a multifactorial interpretation in which the principal late decompensation is attributable to documented upper-airway and aspiration-related mechanisms (confirmed on FEES), with central mechanisms remaining plausible but unquantified in the absence of polysomnography, heart-rate variability testing, and intracranial pressure monitoring.

The possible contribution of secondary hydrocephalus to the post-decannulation hypercapnia cannot be determined definitively from the available data. Serial CT imaging after the new ICH/IVH event suggested mild supratentorial ventriculomegaly compared with the pre-event baseline, but formal quantitative metrics (Evan's index, callosal angle) were not consistently documented, and CSF opening pressure was not measured. The imaging findings are therefore descriptive rather than diagnostic, and the causal relationship between the suggested hydrocephalus and the late rise in PaCO₂ remains hypothetical. Theoretically, chronic communicating hydrocephalus of this magnitude could aggravate brainstem modulatory function through mass effect on periaqueductal and pontine structures, but this hypothesis is not directly supported by intracranial pressure monitoring or by dedicated brainstem imaging in this case. CSF diversion was not performed during the reported course; in retrospect, given the temporal association with the new ICH/IVH and the late decompensation, formal evaluation of CSF dynamics and consideration of CSF diversion would have been appropriate when the imaging first suggested progressive ventriculomegaly.

Several additional candidate mechanisms raised in the recent literature merit consideration in midbrain–pontine hemorrhage. (i) Central sleep apnoea with sustained nocturnal hypoventilation can be associated with brainstem lesions and may contribute to progressive CO₂ retention (14, 15); however, polysomnography was not consistently available in this patient, so the relative contribution of sleep-related events cannot be quantified. The 17-month stable compensated phase suggests that, if central apnoea was present, it was either mild or well-compensated. (ii) Hypothalamic–pituitary–medullary autonomic loops can be disrupted by midbrain injury (16, 17); no clinical signs of hypothalamic–pituitary dysfunction (diabetes insipidus, temperature dysregulation, endocrine abnormality) were documented in this patient, but formal autonomic function testing (heart-rate variability, orthostatic challenge) was not performed. (iii) Weber's syndrome (ipsilateral oculomotor palsy with contralateral hemiparesis) is classically associated with midbrain infarction and frequently accompanied by hypertension and dyslipidemia (18, 19). In this patient, grade 3 hypertension was documented, but no oculomotor palsy or hemiparesis was observed, and a lipid panel was not consistently available during the reported course. (iv) Chronic hypertension can influence cardiorespiratory coupling via carotid body and sympathetic mechanisms (20, 21), but the normal pre-decannulation PaCO₂ argues against chronic hypertensive coupling being the principal driver of late decompensation. (v) Efference-copy signalling between medullary, pontine, and midbrain respiratory centres represents an interesting hypothetical mechanism (22, 23); however, direct neurophysiological testing of efference-copy integrity was not performed. We acknowledge that the available data in this single case do not allow the relative contributions of these candidate mechanisms to be quantified, and we have therefore presented them as plausible but unquantified contributors rather than as established causes.

From a clinical perspective, persistent or rising PaCO₂ after decannulation should prompt reassessment of nocturnal hypoventilation, aspiration burden, airway care, and the feasibility of long-term ventilatory support. Non-invasive ventilation may provide temporary benefit, and existing guidance for chronic hypercapnic respiratory failure supports its use in selected patients (24). However, as illustrated in this case, sustained ventilatory stability may not always be achieved. In patients with recurrent life-threatening hypercapnic decompensation for whom long-term non-invasive ventilation is poorly tolerated, ineffective, or impractical, repeat tracheostomy may represent a reasonable supportive option rather than simply reflecting failed decannulation.

This report has several limitations. As a retrospective single-case report, its findings are not generalizable. Continuous overnight oxygen saturation or carbon dioxide monitoring, polysomnography, and systematic assessment of diaphragmatic or neuromuscular function were not consistently available; therefore, sleep-related hypoventilation and the relative contribution of respiratory pump dysfunction could not be objectively quantified. No MRI of the brainstem was acquired throughout the entire reported course; computed tomography has limited sensitivity for brainstem lesions, particularly in the subacute and chronic phases. Importantly, however, computed tomography performed at the initial onset confirmed hemorrhage confined to the midbrain and pons, with no involvement of the upper medulla (Figure 1a), and follow-up imaging demonstrated cystic encephalomalacia in the same regions. Therefore, an undiagnosed medullary lesion is unlikely to be the principal driver in this case. The bedside clinical signs documented on 28 February 2025, during the same session as FEES—specifically, inspiratory dyspnea with three-concavity sign (suprasternal, supraclavicular, and intercostal retractions), accessory breathing muscle recruitment, stridor, and tachypnea —indicate upper-airway obstruction rather than central hypoventilation. The combination of stridor, accessory muscle use, and dyspnea identifies bilateral vocal cord paralysis, aspiration, and weak cough as the leading cause of respiratory pump failure, which is consistent with the FEES-documented bilateral vocal cord immobility and penetration–aspiration scale level 7 in this patient. In addition, detailed bedside assessment of the cardinal signs of respiratory pump failure—including dyspnea, tachypnea or bradypnea, accessory breathing muscle recruitment, and stridor—was not consistently documented, and serial neurological examination around the time of decannulation was not systematically available, precluding a definitive distinction between central hypoventilation (no dyspnea, no tachypnea) and respiratory pump failure (stridor, accessory muscle use, dyspnea) as the principal mechanism in this patient.

The available data in this single retrospective case do not allow the relative contributions of these mechanisms to be quantified, and formal neurophysiological testing (polysomnography, heart-rate variability, autonomic function testing, intracranial pressure monitoring) would be required in similar patients to support a definitive mechanistic conclusion.

In conclusion, this case suggests that successful decannulation in patients with brainstem injury should not be judged solely on the basis of restoration of anatomical airway patency. The 17-month chronic compensated phase demonstrates that decannulation can be sustained for an extended period when compensatory mechanisms are maintained, and that late decompensation in this setting is more often event-triggered than a manifestation of progressive central drive failure. Ventilatory stability therefore requires longitudinal assessment following tube removal, and although impaired central ventilatory drive was strongly suspected, the clinical trajectory was likely multifactorial. Scheduled carbon dioxide monitoring may enable earlier detection of delayed ventilatory failure in carefully selected high-risk patients—including those with FEES-documented severe aspiration or imaging findings suggestive of hydrocephalus—rather than being applied universally. Pre-decannulation FEES or flexible laryngoscopy should also be considered in every brainstem-injury patient—not only those with documented dysphagia—so that high aspiration risk can be identified before rather than after decannulation. Several clinical recommendations emerge from this case. (1) Every brainstem-injury patient—not only those with documented dysphagia—should undergo pre-decannulation FEES or flexible laryngoscopy, complemented by bedside assessment of upper-airway patency (inspection for inspiratory stridor, accessory breathing muscle recruitment, and three-concavity sign), to identify subclinical vocal cord dysfunction before decannulation. (2) Any patient with suspected acquired central hypoventilation syndrome should have MRI of the brainstem to exclude an undiagnosed medullary lesion, given the limited sensitivity of CT. (3) In brainstem patients, respiratory muscle pump, laryngeal function, cough strength, retention of secretions, and respiratory drive have to be approached holistically with regard to both diagnosis and therapy. (4) Any patient with brainstem stroke undergoing decannulation should be considered high risk, and scheduled transcutaneous or end-tidal CO₂ monitoring during sleep should be considered in the early post-decannulation period. Further studies are warranted to define optimal monitoring protocols and to identify which high-risk subgroups benefit most from targeted surveillance.

## Data Availability

The original contributions presented in the study are included in the article/Supplementary Material, further inquiries can be directed to the corresponding author.
